# Towards a better diagnosis of mouth breathing: validity and reliability of a protocol for assessing the awake breathing pattern in preschool children

**DOI:** 10.1590/2317-1782/20242022330en

**Published:** 2024-04-29

**Authors:** Morgane Warnier, Léonor Piron, Dominique Morsomme, Christelle Maillart

**Affiliations:** 1 Department of Speech-Language Pathology, Research Unit for a life-Course Perspective on Health and Education, University of Liège, Liège, Belgium.

**Keywords:** Children: Preschool, Mouth Breathing, Assessment, Diagnosis, Speech Language Pathology, Psychometrics

## Abstract

**Purpose:**

The Awake Breathing Pattern Assessment (ABPA) is a prototypical clinical grid recently designed through an international consensus of Speech and Language Pathologists (SLPs) to categorize the awake and habitual breathing pattern during the orofacial myofunctional assessment. This cross-sectional study aims to explore the psychometric properties of the ABPA in a preschool population.

**Methods:**

133 children from 2;11 to 6 years old were assessed with the ABPA. The percentage of time spent breathing through the mouth was objectively measured by a CO_2_ sensor and used as a baseline measurement. We first performed a multivariate Latent Profile Analysis based on the CO_2_ measurement and a parental questionnaire to define the number of categories that best characterize the breathing pattern. Subsequently, we assessed the intra- and inter-rater reliability, internal consistency criterion validity, construct validity and sensitivity and specificity.

**Results:**

The awake breathing pattern can best be described by two groups: nasal and mouth breathing. The ABPA, initially designed in three groups, was adjusted accordingly. This final version showed excellent intra-rater and inter-rater reliability. There was a significant correlation between the ABPA and the CO_2_ measurement. The ABPA showed a fair sensitivity and a good specificity.

**Conclusion:**

The reference tool based on CO_2_ data was used in children for the first time and was found to be reliable. The ABPA is a suitable tool for SLPs to confirm the diagnosis of mouth breathing in preschool children if more sensitive screening tools, like parental questionnaires, are used beforehand.

## INTRODUCTION

Screening, diagnosing and treating mouth breathing (MB, also called oral breathing) in the preschool period is a major issue. Nasal breathing (NB) is a prerequisite for the harmonious craniofacial and upper airway development especially before the age of 6^(1)^. However, the diagnosis of MB remains challenging^(2)^ as current tools are not numerous. This is especially true for Speech and Language Pathologists (SLPs).

In most studies, the identification of a MB population regardless of the etiology is based on two elements. First, a set of signs and symptoms that are completely or incompletely present, such as craniofacial features, in the context of a mouth breathing syndrome (MBS)^(3)^. Second, parental questionnaires combining questions related to awake and sleep MB. The most reliable measurements to diagnose sleep MB are those taken by a sensor during polysomnography, for which a pathological threshold is estimated at 15% of the time spent breathing through the mouth^(4)^. A cannula with a sensor has also already been used to identify awake MB in adults in the study by Fujimoto et al.^(5)^ and Nagaiwa et al.^(6)^. This kind of tool is rarely used due to the complexity of its implementation in clinical settings, but has the advantage of being quantitative, objective and directly assessing the breathing function without intermediate information. Other authors use rhinomanometry^(7)^, peak nasal airflow^(8)^ or graded mirror, water retention, and lip seal tests^(9,10)^. Most of these tools are not reliable for the preschool population because the administration is complicated or impossible. Moreover, they all base the identification of MB on nasal airflow resistance/obstruction rather than on the habitual and preferred breathing pattern in everyday life^(11,12)^. While it is imperative to assess and remove the obstruction to rehabilitate nasal breathing, nasal resistance/obstruction is not associated with the breathing pattern^(12)^ and MB does not always result from obstruction. Indeed, functional MB (sometimes called MB by habit^(2)^) is very frequent in children^(13)^.

Most recently, a clinical grid for the Awake Breathing Pattern Assessment (ABPA) was created to address the lack of tools that Speech and Language Pathologists can use to categorize the awake and habitual breathing pattern in the myofunctional assessment^(11)^. Orofacial functions are definitely part of the SLP’s scope of practice^(13,14)^, but the categorization of the breathing pattern still too often relies on the clinical expertise in orofacial myology/myofunctional pathology and the experience with MB patients. This leads to a low agreement between clinicians, as found in other professions^(15)^. To address this, an international panel of experts helped establish a consensus on assessment, allowing the development of a prototype of the ABPA. The international consensus in this study is that breathing should be observed at rest, while chewing and after swallowing, which is congruent with previous data^(13,14)^. The experts also determined that breathing should be classified into three categories (nasal, oronasal and mouth breathing) as suggested by some authors^(5,16)^. Although exclusive MB is extremely rare^(12,17)^, the existence of a separate category for mixed/oronasal breathing (OB) is not unanimous^(18)^. To our knowledge, no study has attempted to answer this question with objective methods.

This current study explores the psychometric properties of the ABPA in the preschool population in terms of construct and criterion validity, internal consistency, intra-rater reliability, inter-rater reliability, sensitivity, specificity and accuracy. To assess the construct validity, we tested the hypothesis of the existence of three categories describing the breathing pattern (NB, OB, MB) using an objective reference measure similar to that described in the study of Fujimoto et al.^(5)^.

## METHODS

### Study design and population

This cross-sectional study was conducted between November 2021 and February 2022 and was part of a larger project on speech and myofunctional development of preschool children. It was approved by the Research Ethics Committee of University of Liège under the protocol B707201940403. Parents of participants gave written consent for their child to participate in the study, authorizing the use of the photos and videos. Children were recruited in kindergarten in the Liège area, Belgium, and were included if they did not present craniofacial anomalies, pulmonary, neurological or cardiac pathologies and/or identified genetic syndromes based on clinical history. We prior estimated the required sample size for the validity analysis using G*Power. A two-tailed correlation test with a moderate effect size (0.3), a power of 0.8, and an alpha of 0.05 requires at least 125 participants. The sample comprised 133 children aged from 2;11 to 6 years old, with a mean of 4;6. The sample included a higher proportion of girls (54.1%) than boys (45.9%).

### Parental questionnaire

Parents were first invited to fill in a written questionnaire on their child’s habits of breathing. We designed a parental questionnaire based on items proved to be discriminating and relevant for the diagnosis of mouth breathing in four studies^(14,19-21)^. Items were selected if they met the conditions for a functional observation of the child’s habitual and awake breathing pattern as previously described^(11)^. We designed a five-point Likert scale ranging from 1 = “never” to 5 = “always” in order to be as comprehensive as possible for the classification of the habitual awake breathing pattern. Items are presented in Table 1.

**Table 1:** Parental questionnaire

During the day, does your child	Never	Hardly ever	Sometimes	Very often	Always
have a blocked/runny nose					
have an itchy nose					
sneeze					
keep the mouth open while engaged in a quiet activity (e.g., watching a movie, drawing, …)					
breathe through the mouth					
appear irritable					
seem sleepy					
tend to eat slowly or to be a picky eater					

### Awake breathing pattern assessment

Children’s awake breathing pattern was assessed by the ABPA and an objective measurement of CO_2_ expired from the mouth which was used as the reference measurement. The second author, assisted by an intensively trained SLP Master student, assessed two children at a time in a quiet room. While one child was assessed with the ABPA, the other was assessed with the CO_2_ tool, and then they switched places. Before the assessment, each child was given a tissue and asked to blow his/her nose.

**Figure 1:**
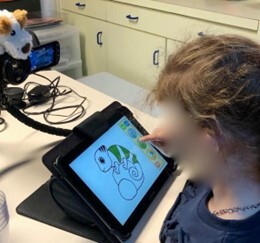
Display of the ABPA assessment

The ABPA includes three main contexts of observation: breathing at rest, breathing after swallowing and breathing while chewing. For the resting items, children were observed while watching a 3-minute cartoon, then a 3-minute coloring and finally another 3-minute cartoon. Activities were displayed on a tablet (Medion Lifetab E10421, Essen, Germany) inclined at 45 degrees. We have selected two silent 3-minute cartoons adapted to children and devoid of any funny parts in order to induce as little speech or laughter as possible. Children were asked not to speak. If the child spoke for more than a few seconds during the 15-minute period, the test was restarted. Resting contexts were interspersed with the swallowing and chewing contexts. Children drank at least three sips of water from a transparent cup and ate a cookie (Speculoos, Lotus®). The whole assessment was recorded with a HD camera (Canon LEGRIA HF G10, Tokyo, Japan) and lasted about 15 minutes. The dispositive is displayed in Figure 1. Scoring of the ABPA was based on the video recordings: three criteria assessed breathing at rest, two assessed breathing after swallowing, and the last one assessed breathing during chewing. Within each criterion, a single sign that described the best the child’s behavior was selected. In the original grid, each sign was associated with a weight coefficient linked to a main and a secondary breathing pattern. Thus, when selecting a sign, its respective weight coefficient and its assigned breathing pattern(s) influenced the final score. In this study, we choose to consider only the main pattern in order to validate this against the percentage of time measurement by the CO_2_. The ABPA was adapted in such a way that only one main breathing pattern would ultimately appear according to the weight coefficient and the pattern linked to the six signs selected. An SLP Master student scored the ABPA on the basis of the recordings. The Master student had previously trained on 15 videos that were not included in this study in order to learn to master the tool, and the reliability of the rating was calculated in relation to the second author's assessment. The inter-rater reliability for the videos of the 133 participants in this study was calculated in comparison to the rating of the first author's rating who has clinical and research experience in the field. Post-scoring of one grid took approximately 5 to 10 minutes.

**Figure 2:**
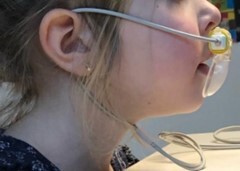
Display of the CO_2_ sensor device measuring expiratory airflow

**Figure 3:**
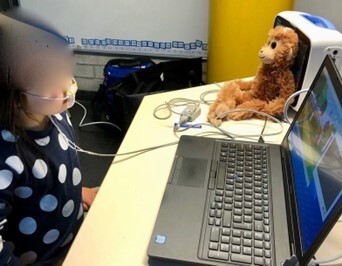
Photograph of the CO_2_ sensor assessment display

On the other hand, the reference assessment consisted in a CO_2_ sensor (Nihon Kohden Cap-ONE Mainstream, Tokyo, Japan) placed on the child’s upper lip while watching a 15-minute cartoon, with similar characteristics to those described above, on a computer. The sensor was originally designed to detect airflow coming from the nose and the mouth. As described in the study of Fujimoto et al.^(5)^, we blocked the nasal tubes of the cannula so that the CO_2_ sensor could only detect the airflow from the mouth. The dispositive is displayed in Figure 2 and Figure 3. Wires connected to the left and right ends of the cannula ran around the ears and held the sensor in place. Children were asked not to touch or move the sensor and not to speak until the cartoon was over. As illustrated in Figure 3, the dispositive was connected to a CO_2_ recording monitor (Nihon Kohden PVM-4000, Tokyo, Japan) which was itself connected to a computer (DELL Latitude 5590, Round Rock Texas, US). The mouth airflow was recorded by a custom-made software called “CO_2_-Analyzer”. The program had been created by PLHealthcare, in collaboration with the University of Liège, for the purpose of the study. The software was launched at the same time as the cartoon. The software instantly transformed the raw data quantifying the amount of CO_2_ exhaled through the mouth to obtain the precise amount of time spent breathing through the mouth (expressed as a percentage). This latter percentage was the final score included in the analyses. A camera (Logitech C920, Lausanne, Switzerland) was placed to the right of the child to synchronously record the face in order to ensure that there was no sensor failure or interruption of monitoring.

The administration time for both tests was on average 30 minutes.

### Statistical analyses

Statistical analyses were performed using R Studio and JAMOVI 1.6.23 software.

Two steps were needed before assessing psychometrics properties of the ABPA. First, we needed to test the hypothesis that awake breathing is best described by three groups (NB, OB and MB), as suggested by the consensus of experts who participated in developing the clinical grid^(11)^. Second, as the continuous variable of CO_2_ percentage was selected as the reference measurement for the psychometric assessment of the ABPA, we needed to discretize it into categories in order to make it suitable for the categorical nature of the grid’s data.

The first step of this process was to determine the number of breathing pattern groups using a multivariate Latent Profile Analysis (LPA). We tested the hypothesis of the existence of two or three groups in the sample. LPA identifies subgroups within a population on the basis of one variable or one set of variables that are conceptually related but distinct. LPA runs several hypothetical groups in the population and determines the best fit based on probabilistic indices. The percentage recorded with the CO_2_ sensor was used as an indicator. Moreover, we decided to add the eight items of the parental questionnaire for more reliability, for a total of nine indicators. Data were either ordinal or continuous variables, which allowed us to use the R package tidyLPA. When conducting an LPA, 6 types of models can be chosen depending on the nature of the mean, variance and covariance of the estimated subgroups. We choose the model 4: a varying variance across profiles and equal covariance. This model was suitable since there was more than one indicator, which automatically induces covariance. We expected equal covariance across profiles as 8 out of the 9 indicators were Likert-scale variables, thus expected to co-vary in a similar way across profiles. We expected varying variance across profiles, as we expected to find variability in the breathing patterns^(12)^. We used the Akaike’s Information Criterion (AIC) and Bayesian Information Criterion (BIC) to detect the correct number of latent profiles. AIC and BIC are the most commonly used information-theoretic methods to select models. They are based on the maximum likelihood estimates of the model parameters, in order to select the most parsimonious and therefore relevant model. Entropy score was considered and we also added the Sample-size Adjusted BIC (SABIC) to correct the sample size penalty induced by the BIC. Finally, we calculated the Bootstrap Likelihood Ratio statistical Test (BLRT). It uses parameters estimation methods to create multiple bootstrap samples to represent the sampling distribution.

The second step of the process was the discretization of the CO_2_ values, which was continuous data, into categories. We discretized the variable from the means and standard deviations (SD) according to the number of profiles obtained by the LPA. These steps resulted in a final version of the ABPA.

Psychometrics properties of the ABPA were assessed with several analyses. Intra-rater and inter-rater reliability were assessed through Cohen’s kappa measurements on 15% of the sample. Internal consistency was measured through a Cronbach’s Alpha.

A Cronbach's alpha (α) <0.7 indicates a lack of internal consistency, α = 0.7-0.9 suggests an adequate internal consistency and α ≥ 0.91 indicates an excellent internal consistency. Interpretation of the agreement based on kappa (k) was as follows: < 0 poor, 0.01-0.20 slight, 0.21-0.40 fair, 0.41-0.60 moderate, 0.61-0.80 substantial and 0.81-1 almost perfect. The percentage of agreement was considered high if greater than 75%, moderate if between 40 and 75% and low if less than 40%. Construct validity was assessed by a known group technique. This analysis assessed the ABPA’s ability to discriminate among the two distinct groups (i.e., NB and MB). The groups were known based on the discretized CO_2_ classification, as it was the reference measurement. A Chi-square test of independence was conducted between groups from the ABPA and from the reference measurement. A significant χ^2^ value indicates that the two distributions are not independent and thus discriminate the groups in a similar manner. Criterion validity was assessed by concurrent validity through a Spearman rank test between the ABPA and the discretized CO_2_. ﻿The correlation coefficient (r) was considered as follows: < 0.19 very weak, 0.2-0.39 weak, 0.4-0.59 moderate, 0.6-0.79 high and 0.8 very high. Finally, a receiver operating characteristic (ROC) curve was designed to assess the sensitivity, specificity and accuracy of the ABPA and its composite scores. The Positive Predictive Value (PPV) gave the probability that children to whom a given breathing mode has been assigned do indeed belong to the given group, while the Negative Predictive Value (NPV) indicates the probability that children who have not been assigned to one group indeed do not belong to that group. The Area Under the Curve (AUC) was calculated. ﻿﻿The AUC quantifies the overall ability of a test to discriminate between 2 outcomes. AUC values range from 0.5 to 1.0. An AUC of 1.0 indicates a perfect test, 0.9–0.99 an excellent test, 0.8–0.89 a good test, 0.7–0.79 a fair test, 0.51–0.69 is a poor test, and 0.5 or less is of no value. Data are available in Supplementary Material 1 .

## RESULTS

### Classification of the breathing patterns

Table 2 shows the descriptive features of the nine indicators that were included in the multivariate LPA in order to determine which classification fitted better between two or three groups. Table 3 shows results from the LPA according to the model with a lower AIC and BIC, a significant BLRT_p and a higher entropy. According to this model, the classification is best described by the existence of two groups.

**Table 2:** Descriptive data of the indicators used the Latent Profiles Analysis

Indicators	N	Mean	Median	Standard deviation	Min	Max
Blocked/runny nose by day	133	2.31	2	0.665	1	4
Itchy nose by day	133	1.59	2	0.640	1	3
Sneezing by day	133	1.95	2	0.535	1	3
Open mouth posture at rest by day	133	2.00	2	0.953	1	4
Mouth breathing by day	133	2.15	2	0.812	1	4
Irritable during the day	133	2.05	2	0.767	1	4
Sleepy during the day	133	1.31	1	0.510	1	3
Slow or picky eater	133	2.28	2	0.948	1	4
Percentage of time spent breathing through the mouth with a CO_2_ sensor	133	24.35	14	25.252	0	85

**Table 3:** Results of the Latent Profiles Analysis

Model	Classes	AIC	BIC	SABIC	Entropy	N_min	N_max	BLRT-p
4	2	3243.58	3428.56	3226.12	1,00	0.29	0.71	0.01*
4	3	3394.33	3608.22	3374.15	0.95	0.30	0.69	0.69

* Statistically significant results

**Caption:** AIC = Aikake’s information criterion; BIC = Bayesian information criterion; SABIC = Sample-size Adjusted BIC; BLRT-p = Bootstrap Likelihood Ratio statistical Test

### Discretization of the CO_2_ percentage

The discretization of the CO_2_ percentage was based on the two groups from the LPA. Values are shown in Table 4. We chose 1.5 SD as a cutoff to discretize the groups, as it better covers the full range of the CO_2_ percentage variable with no overlap between categories. Therefore, scores ranged between 0% and 13.96% for the first profile and between 13.97% and 80.23% for the second profile. The first profile was considered as the NB group the second profile was considered as the MB group. As shown in Table 4, the average percentage of expiratory airflow through the mouth was 7.09% for the NB group and 46.9% for the MB group.

**Table 4:** Mean and standard deviations of the two significant latent profiles from Latent Profiles Analyses

Profile	-2 SD	-1.5 SD	-1 SD	Mean	+1 SD	+1.5 SD	+2 SD
1=NB	-3.85	-1.11	1.62	7.09	12.56	15.29	18.03
2=MB	2.99	13.97	24.95	46.90	68.85	79.83	90.81

**Caption:** NB = nasal breathers; MB = mouth breathers; SD = standard deviation

### Adaptation of the initial clinical grid (ABPA)

**Figure 4:**
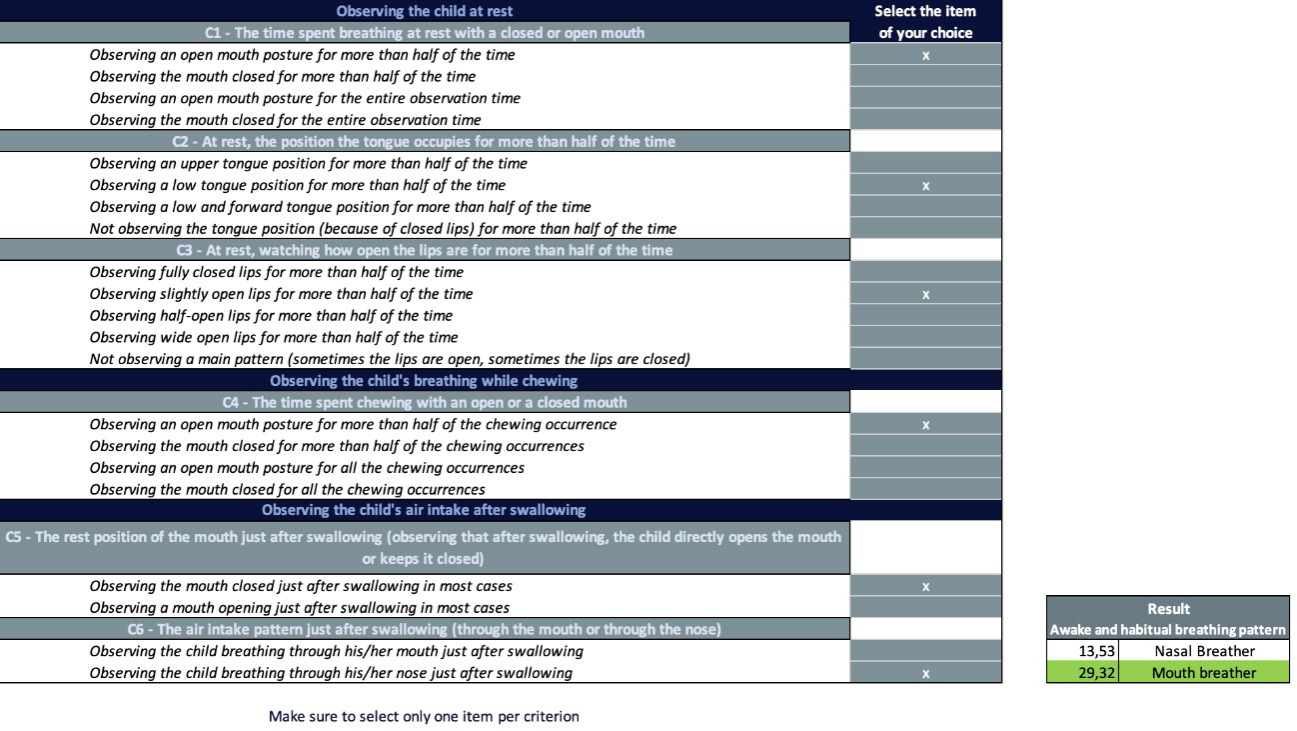
Revised clinical grid (ABPA) including two possible categories of awake and habitual breathing pattern

We modified the prototypical grid according to the LPA results. Originally, there were three groups to classify the awake and habitual breathing pattern. They were merged into two groups, gathering the OB and the MB patterns into one unique group. This revision did not modify any of the original criteria or signs. This revision only affected the signs that were previously associated with the OB pattern. These signs are now associated with the MB pattern. An example of the adapted and final version of the ABPA is available in Figure 4. A blank copy of the ABPA is available in the Supplementary Material 2 for clinical purpose.

### Psychometrics properties of the ABPA

Intra-rater and inter-rater agreement on the ABPA was excellent (96% for both agreements). Cohen’s Kappa was also excellent for intra-rater reliability (k = 0.90, Z = 4.34, *p* <0.001) and inter-rater reliability (k = 0.92, Z = 4.50, *p* < 0.001). Internal consistency of the grid was good (Cronbach’s Alpha = 0.85).

According to the grid, 83 children were classified as NB and 50 children were classified as MB. According to the CO_2_ measurement, 74 children were classified as NB and 59 children were classified as MB. The chi-square test of independence showed a significant association between the classification results of the ABPA and the discretized CO_2_ (χ^2^ = 28.5, df = 1, N = 133, *p* < 0.001). A moderate but significant Spearman correlation was found between the two classifications (r = 0.46, N = 133, *p* < 0.001).

**Figure 5:**
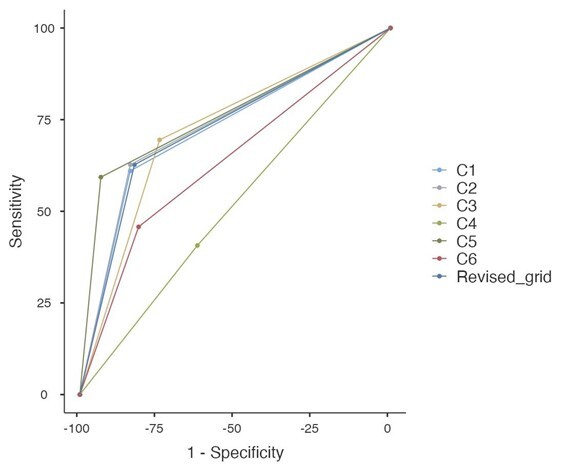
ROC curve between the ABPA, its composite scores and the CO_2_ percentage

We finally assessed the discriminant validity and accuracy of the ABPA and each criterion of the grid, in comparison to the CO_2_ measurement. Table 5 summaries the results of the sensitivity, specificity, PPV, NPV and AUC of the composite and total score. Figure 5 displays the ROC curves showing that the distinction between NB and MB according to the ABPA was not due to chance (AUC = 0.726 > 0.5). The revised ABPA was a fair test (0.7 < AUC < 0.9).

**Table 5:** Discriminant features of the total score and composite items of the ABPA compared to the CO_2_ measurements

Scale	Sensitivity (%)	Specificity (%)	PPV(%)	NPV(%)	AUC
Total Score	62.71	82.43	74	73.49	0.73
C1 - The time spent breathing at rest with a closed or open mouth	61.02	83.78	75	72.94	0.72
C2 - At rest, the position that the tongue occupies for more than half of the time	62.71	83.78	75.51	73.81	0.73
C3 - At rest, watching how open the lips are for more than half of the time	69.49	74.32	68.33	75.34	0.72
C4 - The time spent chewing with an open or a closed mouth	40.68	62.16	46.15	56.79	0.51
C5 - The rest position of the mouth just after swallowing (observing that after swallowing, the child directly opens the mouth or keeps it closed)	70	93.98	87.5	83.87	0.82
C6 - The air intake pattern just after swallowing (through the mouth or through the nose)	45.76	81.08	65.85	65.22	0.63

**Caption:** PPV = Positive Predictive Value; NPV = Negative Predictive Value; AUC = Area Under the Curve

## DISCUSSION

The first step to verify the psychometric properties of the grid was to check its validity in comparison to a reference tool. This type of objective tool is being used for the very first time for this purpose in children. This measurement has proven to be an objective, reliable, relatively easy to use measure despite some constraints specific to the pediatric population. First, the only cannulas designed for children that are available with the device used are intended to newborns. The adult cannulas used were therefore relatively large for the young children in the sample, but this only affected the child's comfort. The second constraint was that young children initially tended to touch the cannula or speak despite prior instructions not to do so, which was not encountered in the highly controlled adult experiment in the study of Fujimoto et al.^(5)^. To address this and minimize the effects on the recording, we took care to remind the child immediately of the instruction. Moreover, the classification method used allowed us to take into account and homogenize this factor. This classification method, which also takes into account the parental questionnaire, allowed us to classify breathing into two distinct patterns: NB *versus* MB. This goes against the international consensus established by the previous study^(11)^ and supported by some authors^(5,18,22)^. This consensus was based on expert’s opinion and therefore remained subjective. The results of this study allow us to consider OB as a mild form of MB but not as a distinct pattern. This postulate is supported by some authors^(12,14,19,23)^. Yet, the high variability found in this study within the MB group suggest that the breathing pattern is best described as a general and predominant trend^(16,17)^ and that MB should be viewed as a continuum within which exclusive mouth breathing is rare^(12)^. This is probably why OB was considered clinically relevant as a buffer zone between MB and NB. Anyhow, the authors who classify OB as a separate category agree that OB should be considered as a pathological condition^(22)^, which can actually be taken as an argument for the existence of two categories. Our results are also congruent with the study of de Mattos et al.^(24)^ which shows that the muscular characteristics of the OB and MB groups are similar, but differ significantly from group NB. Further studies exclusively based on objective measures are now needed to compare the clinical features between the OB and the MB groups to determine once and for all whether it is worthwhile to separate these categories. Overall, this two-tailed classification might bring new advancements in the definition of MB and may clarify the clinical interest of OB. Interestingly, a small but non-null variability was observed within the NB group: air expired through the mouth varied from 0% to 13% of the time for these children. These results differ from the classification used by Fujimoto et al.^(5)^ who considered NB as strictly equal to 0% in adults. This variability could therefore be explained by the specific constraints of the preschool age mentioned above. In contrast, studies using similar methods to diagnose sleep MB found that nasal breathers spend approximately 0-10% of the sleep time breathing through the mouth^(4)^, as does our population.

Both tests were similar in that they observed the breathing pattern at rest and the administration was sequential. Same day administration is important as there is an inherent fluctuation in breathing^(12)^. However, there were also differences between the tests. The ABPA included a coloring activity which required motor activity. In some cases, we observed that the motor activity put the mouth under tension and changed the mouth posture to accompany the hand gesture. This observation is congruent with the synergies observed between the motor movements of the hand and mouth^(25)^. Despite this, we believe, as did the experts of the international consensus, that various observation contexts are more representative of the natural and daily functioning of the child^(11)^. Another difference between these tests is related to the head posture during the administration. Indeed, for the CO_2_ experiment, the computer was presented at a 90° angle, encouraging the child to hold his head upright. For logistical reasons, the ABPA activities were presented on a tablet inclined at 45°. This tilt may have caused anterior flexion of the head and changed mouth posture as muscle chains and breathing are strongly connected^(26)^.

The classification of the breathing pattern according to two profiles made it possible to adapt the initial ABPA grid. Modifications and improvement are typically part of the elaboration process^(27)^. The final version of the grid (differentiating between a NB profile and a MB profile) appears to be valid and accurate in its use by SLP in the myofunctional assessment context. The tool requires a short training period to learn how to use it, but the excellent inter-rater reliability shows that the results depend on tangible observations rather than on the expertise of the clinician. It can therefore be assumed that even SLP with little experience in the field of myology/myofunctional science will be able to use it. Once the SLP is familiar with the tool, scoring can be done at the same time as administration, reducing its use to 15 minutes. The swallowing and chewing tests included in the administration of the grid could be used as a basis for analyzing the quality of swallowing and chewing functions. This would reduce the administration time in a comprehensive assessment of orofacial functions.

The ABPA shows a good specificity but lacks sensitivity compared to our reference tool. The grid can therefore be used to confirm the breathing pattern but is less effective in screening children with MB. The comparison of a subjective grid to an objective reference measurement may explain its low sensitivity. The reference measurement refers to strict categories based on a cutoff score that allows little flexibility, whereas the grid is less rigorous because it allows for a certain gradation. The choice of cutoff score to discretize the continuous data into categories therefore may have affected the sensitivity^(28)^. We have chosen to divide the measures into two groups which cover the full range of the CO_2_ percentage variable with no overlap between categories. By choosing another cutoff, the sensitivity could have been different but at the expense of the specificity^(28)^. The good specificity makes the ABPA a good confirmatory tool^(29)^, showing its usefulness to confirm the diagnosis of awake MB regardless of the etiology. This fulfills the tool's primary purpose as the ABPA was designed to be part of the myofunctional assessment, most often coming as a second line. The ABPA should however require the prior use of more sensitive screening tools to suspect MB, such as questionnaires^(19)^.

The ABPA could be particularly useful for the SLP to confirm MB suspected by other practitioners. We strongly believe that professions are complementary, but have different roles to play in the assessment and diagnosis of MB. SLPs need to adopt a functional viewpoint, while dentists and orthodontists have to consider a morphological and dental viewpoint, with the physiotherapist considering the whole body posture and the ENT specialist having an crucial role in determining whether the cause is obstructive or not. Each profession must therefore develop its own tools and validate them according to its own objectives. This is why the ABPA is not intended to determine the cause of MB, as the ENT examination is essential for this purpose. On the other hand, the ENT examination alone cannot determine the child's habitual breathing mode in daily life; the myofunctional examination is essential for this. The roles are different, but complementary. A multidisciplinary approach remains therefore essential^(13)^.

Finally, it is important to keep in mind that the challenge of MB diagnosis lies in the notion of chronicity^(30)^. The breathing pattern could change over time. For this reason, it seems adequate to administer the ABPA at various intervals, in order to better assess the chronicity of the breathing pattern.

## CONCLUSION

The final version of the ABPA has good construct validity, excellent intra-rater and inter-rater agreement, fair accuracy and good specificity but lacks sensitivity to identify mouth breathing in preschool children.

The objective reference tool based on CO_2_ is a very promising research method for SLP, but also for dentists, orthodontists, otorhinolaryngologists or physical therapists. The use of an objective reference tool to assess breathing in young children demonstrates that it is possible to select a sample of the study population on the basis of objective data, which is necessary for future scientific research. It may also allow, as it has been done here, the validation of clinical tools that each profession uses to classify the awake breathing pattern or used to objectively estimate the prevalence of MB.

In conclusion, the final version of the ABPA, can be reliably used in the SLP's myofunctional assessment to quickly confirm the breathing pattern of preschool children based on well-defined contexts, criteria and signs. We encourage further projects on the ABPA to improve its sensitivity in assessing the young population and make its use more versatile. Further studies are now needed to assess the psychometric properties in populations of different ages or with associated medical conditions.

## Supplementary Material

Supplementary material accompanies this paper.

Supplementary Material 1

Supplementary Material 2

This material is available as part of the online article from https://doi.org/10.1590/2317-1782/20242022330en
